# ZnO doped PAMAM for asphalt improvement as anti-corrosive coatings

**DOI:** 10.1038/s41598-024-78875-5

**Published:** 2024-11-16

**Authors:** Abdalrahman G. Al-Gamal, Walaa S. Gado, Muhammad A. Abo El-Khair, Khaled Zakaria, A. A. Ragab, Khalid I. Kabel

**Affiliations:** Egyptian Petroleum Research Institute (EPRI), Nasr City, Cairo, 11727 Egypt

**Keywords:** Anti-corrosive coating, Asphalt binder, Hyperbranched polymer linker, EIS, Electrochemistry, Corrosion, Polymers

## Abstract

Asphalt is widely used as a coating resin due to its excellent adhesion strength and cost-effectiveness; however, its limited corrosion protection necessitates enhancement. In this study, poly(amidoamine) (PAMAM), combined with zinc oxide (ZnO) nanoparticles, was incorporated into the asphalt matrix to improve its anticorrosive properties. Various ratios of PAMAM-ZnO nanocomposite (1, 2, 4, and 6% by weight) were added to the asphalt binder, with the materials characterized using XRD, ¹H-NMR, and SEM techniques. The 2% PAMAM-ZnO/asphalt ratio exhibited the most significant improvement, achieving a corrosion protection efficiency (η%) of 97.93%, as confirmed by Tafel analysis, and a charge transport resistance (R_CT_) of 75.91 Ω cm² according to electrochemical impedance spectroscopy (EIS) data. A combination of barrier formation and sacrificial protection drives the corrosion inhibition mechanism. The PAMAM-ZnO nanocomposite forms a highly uniform layer on the carbon steel surface, creating an effective physical barrier that prevents the penetration of corrosive agents, thereby minimizing defects like pinholes. This barrier effect is complemented by the sacrificial protection provided by the ZnO nanoparticles, which are more reactive than the underlying steel and preferentially interact with corrosive ions (e.g., chloride ions). This interaction leads to the formation of stable ZnO corrosion products, which enhance the barrier and reduce the likelihood of corrosion on the steel surface. Additionally, PAMAM facilitates the even distribution and strong adhesion of ZnO within the asphalt matrix, ensuring a durable protective layer. The synergic impact between the polymer barrier and sacrificial ZnO protection results in the exceptional corrosion resistance observed in the 2% PAMAM-ZnO/asphalt formulation, offering a promising approach for advanced anticorrosive coatings.

## Introduction

Metallic surfaces are often shielded from severe corrosion through the application of protective coatings, which operate via two primary mechanisms: the barrier effect and the sacrificial effect^1^. The barrier effect functions by forming a continuous, impermeable layer that either physically blocks or significantly prolongs the diffusion pathways of corrosive species such as chloride ions (Cl-), oxygen radicals (O•), or hydroxyl ions (OH-) from reaching the underlying metal surface. This mechanism relies heavily on the coating’s integrity, thickness, and the homogeneity of the applied layer. Even minor defects or pinholes can compromise the coating’s effectiveness, allowing corrosive agents to permeate and initiate localized corrosion^2^.

On the other hand, the sacrificial effect is rooted in electrochemical principles, where specific additives within the coating preferentially undergo oxidation, thereby consuming corrosive species and protecting the metal substrate from direct attack. These sacrificial additives, typically more anodic than the substrate, serve as the primary sites for corrosion, gradually degrading while sparing the metal surface^2^. The choice and integration of such additives are critical to ensure their effective dispersion and interaction within the coating matrix.

Asphalt is extensively utilized as a coating resin due to its intrinsic properties, including superior adhesion, mechanical robustness, and cost-efficiency^3^. However, its application in anticorrosive systems is hampered by inherent limitations such as suboptimal barrier performance, extended curing times, and inadequate film hardness, which collectively diminish its long-term efficacy as a protective coating^4,5^. Extensive research has focused on augmenting asphalt with various modifiers, including metal oxides^6^, graphene derivatives^7^, organic compounds^8^, and advanced polymers^9^, to overcome these deficiencies. These additives are incorporated to enhance the asphalt matrix’s physicochemical properties, aiming to improve its resistance to environmental stressors and corrosive agents.

Recent advancements in polymer chemistry have ushered in a new era of materials, particularly dendrimers^10^ and hyperbranched polymers (HBPs)^11^, which have garnered significant attention due to their unique structural characteristics and potential applications in coatings^12^. Among these, poly(amidoamine) (PAMAM) dendrimers have emerged as a versatile class of materials owing to their highly branched, tree-like architecture that endows them with a multitude of reactive end groups^13,14^. The hyperbranched structure of PAMAM dendrimers offers a high density of functional sites, facilitating strong interactions with both the coating binder and the dispersed nanoparticles. This structural configuration not only enhances the mechanical properties of the coating but also contributes to the formation of a densely packed, hydrophilic/hydrophobic interface, which can significantly impede the ingress of water and corrosive ions^15^. Additionally, the PAMAM dendrimers can serve as a molecular crosslinker, promoting the formation of a more cohesive and resilient coating matrix.

Empirical studies have demonstrated that integrating PAMAM into coating formulations can result in significant rearrangement of the binder’s internal matrix, thereby enhancing the coating’s barrier properties. This rearrangement is attributed to the ability of PAMAM to interact with various components within the matrix, leading to a more compact and less permeable structure that effectively repels corrosive species from the metal surface^16,17^. However, it is well-recognized that while PAMAM enhances the coating’s barrier properties, it does not provide active corrosion inhibition once corrosive agents breach the coating^18^. This limitation underscores the need for the strategic incorporation of additional nanoparticles that can engage in sacrificial protection, thereby offering a dual mechanism of defense against corrosion^19^.

Zinc oxide (ZnO) has been widely employed as a corrosion inhibitor due to its favourable electrochemical properties and its ability to act as a sacrificial agent within protective coatings^20,21^. ZnO nanoparticles (ZnO-NPs) exhibit a high surface area-to-volume ratio, which enhances their reactivity and interaction with corrosive species. When integrated into a coating matrix, ZnO-NPs contribute not only to the sacrificial protection but also to the mechanical strengthening of the matrix by improving its modulus and hardness^22,23^. Furthermore, ZnO-NPs have been shown to synergistically enhance the barrier properties and electrochemical performance of polymer-based coatings, particularly when used in conjunction with materials like PAMAM^24–26^. The combined use of ZnO and PAMAM in a coating matrix leverages the structural benefits of PAMAM with the reactive, sacrificial capabilities of ZnO, providing a robust defense against corrosion.

For example, M. Ramezanzadeh et al. explored the use of PAMAM dendrimer-reduced graphene oxide nanosheets (GO-PAMAM) in an epoxy binder, developing a high-performance anti-corrosion coating. The study demonstrated that PAMAM molecules, when grafted onto the GO surface, increased lamellae d-spacing and improved particle dispersion within the epoxy matrix. This structural enhancement contributed to the coating’s superior corrosion protection, as evidenced by electrochemical impedance spectroscopy (EIS) and salt spray testing^16^.

In this study, we investigate the influence of incorporating PAMAM-ZnO into an asphalt matrix on the corrosion protection of carbon steel (CS) surfaces. The PAMAM-ZnO composite was meticulously characterized using scanning electron microscopy (SEM), X-ray diffraction (XRD), and proton nuclear magnetic resonance (¹H-NMR) analyses. The impact of PAMAM-ZnO nanoparticles on the corrosion protection performance of the asphalt coating was evaluated through electrochemical impedance spectroscopy (EIS) and Tafel polarization studies. The Nyquist and Bode plots were fitted to an electrical equivalent circuit (EEC) to elucidate the electrochemical behavior of the coatings. The data revealed that the optimal ratio of 2% PAMAM-ZnO/asphalt provided superior corrosion protection for CS, significantly outperforming other tested ratios. This finding underscores the potential of PAMAM-ZnO as an advanced additive for enhancing the anticorrosive properties of asphalt-based coatings.

## Experimental

### Materials

Zinc sulfate heptahydrate (ZnSO_4_.7H_2_O, Sigma-Aldrich, 99%), sodium hydroxide (NaOH), H_2_O_2_, ethanol, Methyl acrylate (MA, Sigma-Aldrich, 99%), (3-aminopropyl) triethoxysilane (APTES, Sigma-Aldrich, 99%), and ethylene diamine (ED, Merck, 99%), Benzoyl peroxide (BP) was supplied from Merck, n-hexane, ethanol, and methanol were purchased from Merck Company—Egyptian asphalt penetration grade (60/70) produced by Al-Nasr Petroleum Company in Suez, Egypt.

### Methodologies

#### ZnO nanoparticles Preparation

Zinc sulfate heptahydrate (ZnSO_4_.7H_2_O) 0.1 M solution and sodium hydroxide (NaOH) 0.4 M solution were prepared separately. These solutions were mixed in a round-bottom flask and stirred for 15 min at room temperature. Subsequently, the round-bottom flask was placed in the microwave for 2 min to complete the reaction. After that, the reaction flask was cooled to room temperature, separated via filtration, thoroughly washed with deionized water (DW), and dried at 40 °C. The obtained product is ZnO-NPs.

#### Modification of ZnO-NPs with APTES

In a round-bottom flask, 3 g of the prepared ZnO-NPs were dispersed in 500 mL of H_2_O_2_. The mixture was sonicated for 24 h in a water bath sonicator. The sonicated ZnO-NPs were washed with DW and subsequently dried at 60 °C for 24 h to obtain oxidized ZnO-NPs. 2 g of oxidized ZnO were dispersed in 500 mL of ethanol in a three-nicked flask, followed by sonication. After sonication, an appropriate quantity of APTES was added to the mixture. The reaction mixture was then maintained at 80 °C for 24 h in an oil bath equipped with a reflux condenser. After the reaction was completed, the mixture was filtered and washed using DW. The obtained ZnO-APTES were dried in a vacuum oven at 60 °C for 12 h. Modifying ZnO with APTES to form ZnO-APTES is a strategic approach to enhance the versatility and functionality of ZnO nanoparticles, leading to improved interaction between the ZnO nanoparticles and the PAMAM matrix. The amino groups introduced by APTES enable further chemical modifications, improve compatibility, better dispersion, and stronger chemical bonding with the polymer, and enhance the overall chemical and physical properties of the ZnO nanoparticles. Also, it can reduce the tendency of ZnO nanoparticles to agglomerate by providing steric hindrance and electrostatic repulsion. This leads to a more stable dispersion of nanoparticles in various media.

#### In-situ polymerization of PAMAM-ZnO nanocomposite preparation

**Scheme 1:**
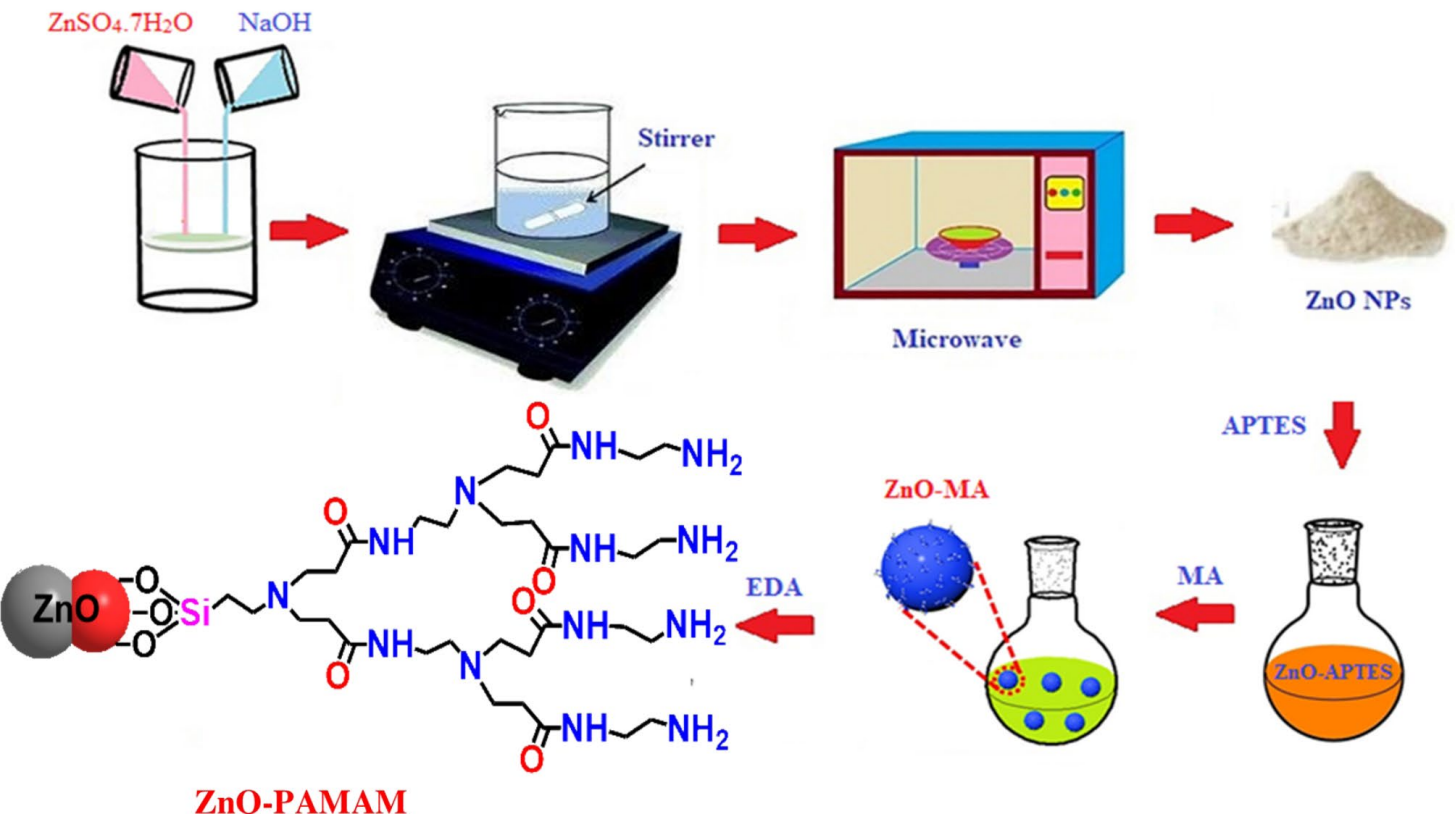
The schematic steps of ZnO-PAMAM preparation.

In a three-nicked round flask, a solution of (100 mg) ZnO-APTES and (30 mL) methanol was subjected to ultra-sonication for 30 min. This ensures a homogeneous distribution of ZnO-APTES in the solvent and enhances the reactivity of the surface. Then, (1.6 mL) of methyl acrylate dissolved in (5 mL) methanol was added dropwise at 0^◦^C under continuous stirring and nitrogen atmosphere for 3 h. This step initiates the surface modification of ZnO-APTES, introducing acrylate groups onto the ZnO surface. Then, the reaction was kept stirring at room temperature for 3 days to allow the complete attachment of MA to the ZnO surface. After that, the sample was put in a rotary evaporator to obtain the final product ZnO-MA. This step was repeated three times after washing the final product with ethanol, then n-hexane was used to precipitate ZnO-MA and dried in the oven at 60^◦^C for 24 h. A (0.2 g) ZnO-MA solution in (30 mL) methanol was subjected to ultra-sonication for 30 min. Then, slowly add the solution of (4.75 mL) ethylenediamine and (50 mL) methanol in a nitrogen-purged flask at 0◦C, stirring for 4 h., which initiates the in-situ polymerization process, where ethylenediamine reacts with the methyl acrylate groups to form amide bonds for the PAMAM dendrimer structure. The mixture was stirred at room temperature for 3 days, allowing the PAMAM dendrimers to grow on the ZnO surface. The final product was centrifuged at 6000 rpm and washed with ethanol. Finally, oven drying was used for 48 h at 50^◦^C to produce ZnO-PAMAM, see Scheme 1. Further details can be found in the previously published study^27^.

### Materials and coating films characterizations

The prepared polymer was characterized using different techniques such as Fourier transform infrared (FTIR) spectra, the particle size distribution was measured by dynamic light scattering (DLS), X-ray diffraction (XRD), nuclear magnetic resonance (^1^H- NMR), and scanning electron microscope (SEM). FTIR spectra were recorded on a NICOLET 5,700 infrared spectrometer (Thermo Nicolet Corporation, USA). XRD was performed using X’Pert Pro, Malvern Co., UK, with swapping rang 2θ: 5° − 90°, λ = 1.5406 Å. NMR spectra were recorded on a Bruker 400 MHz (Varian, USA) using dimethyl-d6 sulfoxide (DMSO-d6) as solvent. GPC measurements were carried out on a Waters 1,515 instrument referenced with polystyrene as standards and N, N-dimethylformamide (DMF) as solvent. SEM was measured by S-4700 scanning electron microscope. The Dynamic Light Scattering (DLS) investigated the particle size distribution with a laser angle 90° at 25 °C (Zetasizer Nano-ZS90 instrument, Malvern Co., UK).

### Characterization of unmodified asphalt

The sample of original asphalt tested as described in ASTM D36 softening point^28^, (ASTM D5) penetration^29^, ASTM D4402 Brookfield viscosity^30^, ASTM D3297 n-heptane insoluble^31^, and specific gravity (ASTM D70)^32^. The obtained results are exhibited in Table 1. The temperature susceptibility of virgin and modified asphalt samples was expressed in terms of penetration index (PI) using the penetration (at 25 ^0^C) and softening point values. PI can be calculated from the following equation^33^:

1 $$\:P.I.=\:\frac{1952-500\:{\text{log\:}(Pen}_{25})-20\:Softening\:Point}{50\:{\text{log\:}(Pen}_{25})-\:Softening\:Point}$$

Pen 25 = penetration value at 25 ^0^C, 0.1 mm & SP softening.

**Table 1:** Physical characteristics of unmodified asphalt sample.

Test	AC	SP*
Penetration (@ 25ºC, 100 g, 5 s), 0.1 mm Softening point (ring and ball), ºC Penetration index Specific gravity (@ 25ºC) using a pycnometer, Flashpoint (Cleveland open cup), ºC. Kinematic viscosity (@ 135ºC), cSt. Thermal Gravimetric analysis (TGA) *** Initial degradation temperature, ºC. *** Final degradation temperature, ºC. *** Total weight loss (wt%). Chemical composition Maltene (wt %) Asphaltene (wt %)	64 50 −0.40 1.02 300 335 246.0 520.0 85.14 73.8 23.6	60/70 45/55 −2: +2 NS** + 250 > 320 NS** NS** NS**

N.B.

* Standard specification for ‘‘General Authority for Roads, Bridges and Land Transportation in Egypt”, Item No 102.1.

** Not specified.

*** According to the literature^34^, the maximum difference is 2.

### Asphalt/ PAMAM-ZnO composite coatings preparation

The coatings were prepared by utilizing commercial asphalt as a coating binder. The physical and chemical specifications of this binder are detailed in Table 1. The PAMAM-ZnO composite was finely ground in a porcelain dish to ensure uniform dispersion and eliminate agglomerations. Consequently, PAMAM-ZnO particles were obtained in the size range of 1 nm to 1 μm. In-situ pristine asphalt polymerization and four PAMAM-ZnO composite levels (1, 2, 4, and 6% wt of asphalt) were prepared in suitable cans. Then, a high-shear mixer was dipped into the sample and set to 3000 rpm. The composite was added gradually (5 g/min) with 0.5 to 1.0 gm BP as initiator. The temperature was kept within 180 ^0^C during the polymer addition and subsequent mixing. Then, stirring was performed for 2 h after the complete addition of polymer, initiator, and cross-linking agent with a fixed amount of 2 ml into asphalt^35^. Under high temperatures, partial active groups of PAMAM-ZnO molecules were reacted with active bonds in asphaltene.

**Scheme 2:**
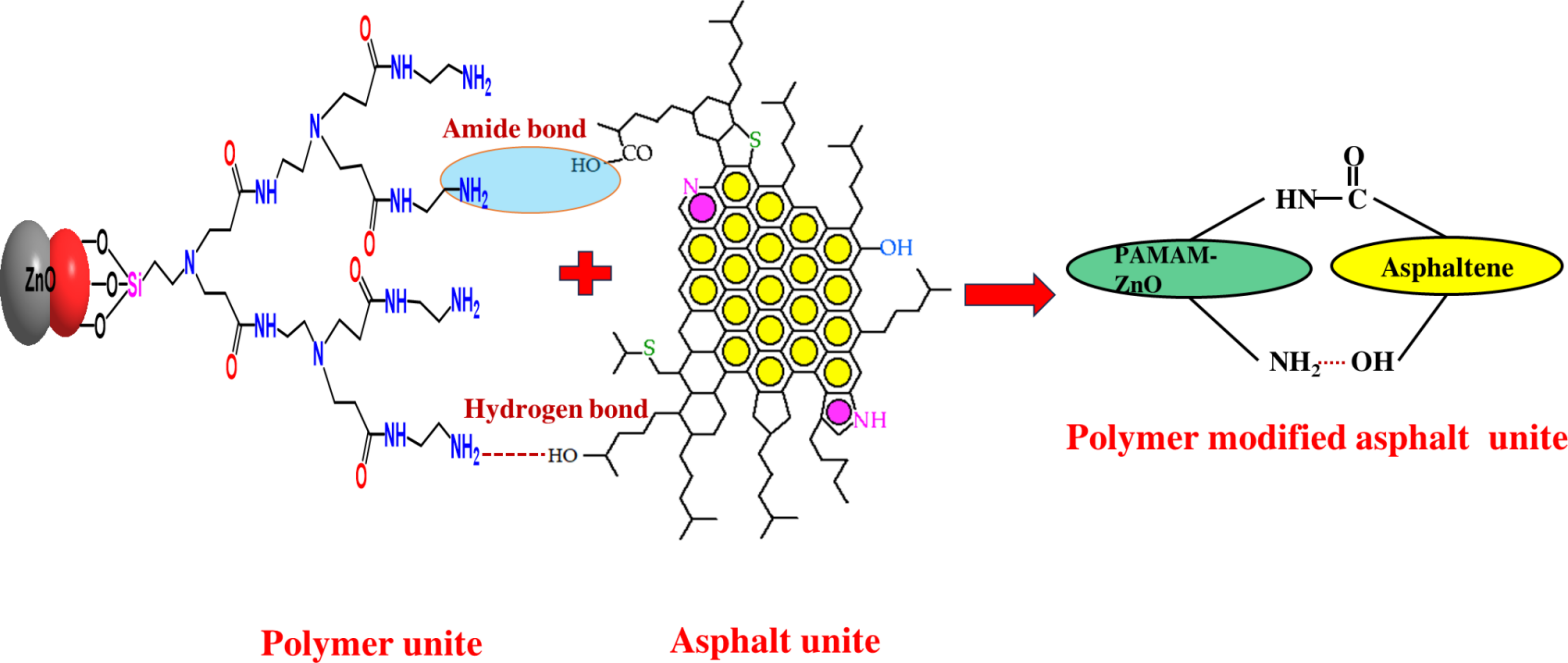
The possible bond formation between ZnO-PAMAM nanoparticles and asphaltene molecules.

Moreover, an asphaltene molecule, or micelle, contains more than one carboxylic group, so a chemical network theoretically will form. Unfortunately, due to the highly complex chemical nature and composition of asphalt, it is difficult to detect the real nature of chemical bonds formed during cross-linking. Scheme 2 illustrates the possible bond formation between ZnO-PAMAM nanoparticles and asphatene molecules in the maltene matrix. It represents the schematic reaction of in-situ polymerization of PAMAM-ZnO and asphalt. Finally, all the formulated coatings were further diluted with a 50% weight% of xylene solution, serving as a solvent to make it easier to apply onto the carbon steel surface.

### Metal surface pre-preparation and applying the prepared coatings

Carbon steel specimens with dimensions 1 cm × 10 cm× 1 mm were readied following the guidelines laid out in ASTM G31. The chemical composition of the carbon steel alloy is outlined in Table 2. These samples underwent a process that involved polishing with an iron emery motor and smoothing with emery papers (1200 grit). Afterward, they were meticulously cleaned and degreased through ultrasonication before being stored in a dry environment. The prepared formulations were then applied to the carbon steel using a film applicator, resulting in an average thickness of 45 μm. Subsequently, the coated steel was allowed to air dry at 24 °C for 2 days.

**Table 2:** Chemical composition of carbon steel.

Element	Fe	C	Si	Mn	Mo	*P*	S	Ni	Cr	Cu
(W %)	96.12	0.13	0.42	1.58	0.74	0.04	0.03	0.31	0.31	0.33

### Evaluations of the prepared formulations as anti-corrosive coatings

#### Rheology of formulated coatings

The Brookfield DV-III Ultra Programmable Rheometer, following the ASTM D4287 (1994) standard, is used to measure fluid properties like Shear Stress and Viscosity at specific Shear Rates. Viscosity quantifies a fluid’s resistance to flow. The device operates by rotating a spindle submerged in the fluid using a calibrated spring. The fluid’s resistance causes the spring to deflect, and a rotary transducer records this deflection. The viscosity ranges the DV-III Ultra can measure in centipoise (c.P) vary with the spindle’s rotational speed. This method is applied to both unmodified asphalt and asphalt samples containing four levels of PAMAM-ZnO composite (1, 2, 4, and 6% by weight of asphalt).

#### Abrasion resistance evolution

The abrasion test method measures the resistance of organic coatings to wear using the Taber Abraser, as outlined in ASTM D 4060 (1995). Coatings are applied uniformly to a flat, rigid surface like a metal panel, and after curing, the surface is abraded by rotating it under weighted abrasive wheels. Abrasion resistance is determined by the weight loss after a specified number of cycles, the weight loss per cycle, or the number of cycles needed to remove a unit thickness of the coating. In this study, the ABRASER 325 model was used for testing.

#### Electrochemical measurements

Electrochemical experiments were conducted using conventional cells equipped with three electrodes to evaluate the prepared formulations as protective coatings for carbon steel under an aggressive corrosive medium (1 M HCl solution). The three-electrodes cell was operated by an Origalys signal multi-channel instrument connected to a computer software-controlled electrochemical process (OrigaFlex 01 A). In this setup, the working electrode (WE) was coated with different formulated coatings, and the exposed area to the corrosive medium was 1 cm^2^. The saturated calomel electrode (SCE; Ag/AgCl saturated with KCl) was utilized as a reference electrode (RE), and a rectangular platinum foil with a 1 cm^2^ surface area as the counter (auxiliary) electrode (CE).

The electrochemical investigations encompassed several techniques, including open circuit potential (OCP). Other tests are Tafel polarization and electrochemical impedance spectroscopy (EIS), which were carried out when the tested electrode reached a steady state of OCP. In the initial stage, we recorded the open circuit potentials (OCP) for 15 min. Subsequently, the WE underwent polarization in both cathodic and anodic directions. The polarization curves were obtained by automatically varying the electrode potential from − 700 mV to -300 mV, employing a scan rate of 2 mV/s. Impedance measurements were conducted by applying a frequency range spanning from 100 kHz down to 200 mHz, with 20 steps per frequency decade. A peak-to-peak AC signal with an amplitude of 150 mV was employed to perturb the system. The recorded Nyquist plot was subjected to fitting using Zsimwin Version 3.0.1 software to further analyze the impedance data. This allowed us to derive an electrical equivalent circuit (EEC) with satisfactory errors lower than 10%. Every test was repeated 3 folds for repeatable and reproducible.

## Results and discussion

### Materials characterizations

**Fig. 1:**
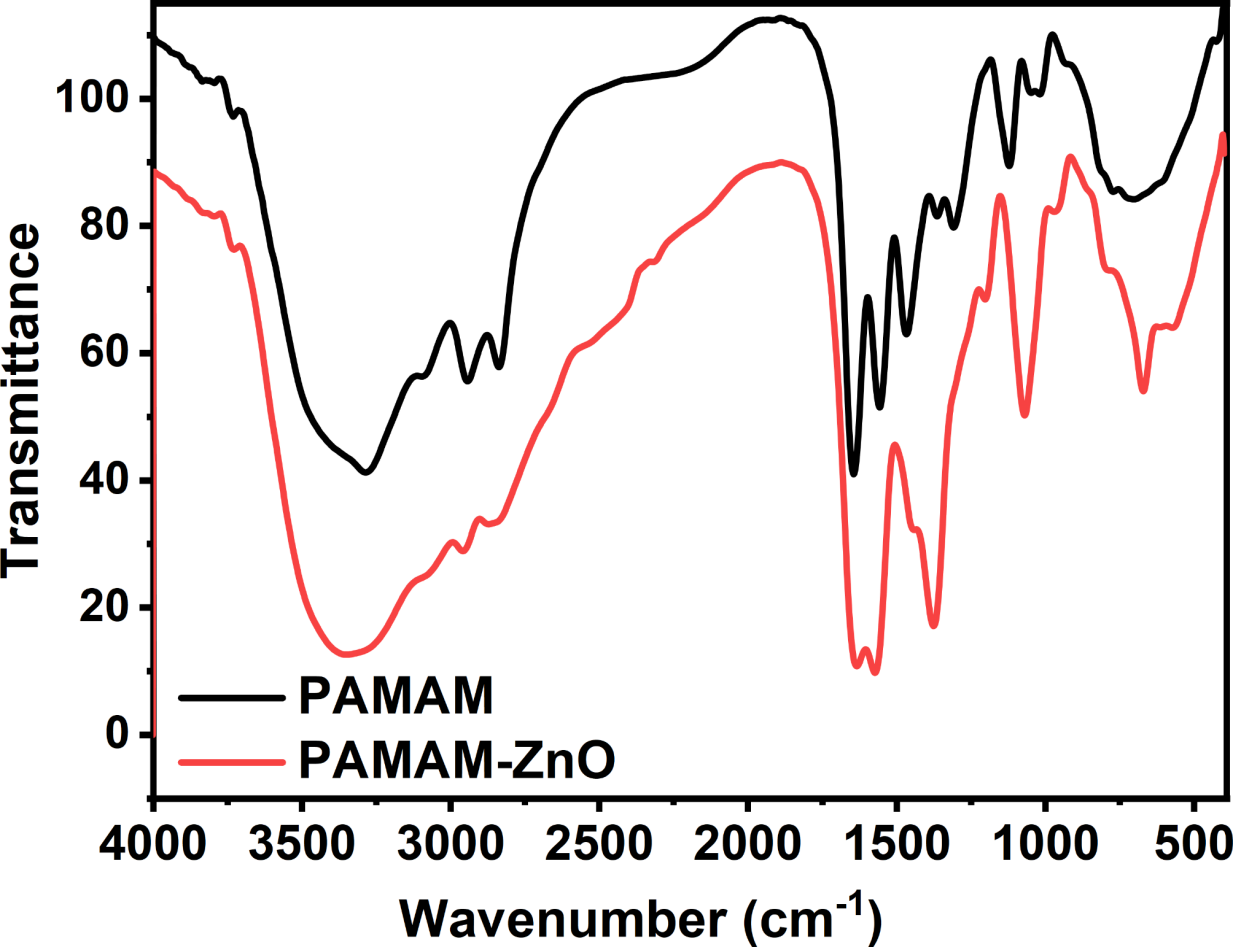
FTIR spectrum of PAMAM-ZnO.

The FT-IR spectroscopic analysis of the prepared materials is shown in Fig. 1. The black and red curves represent the transmittance of PAMAM and PAMAM-ZnO, respectively, which confirmed the presence of vital functional groups within the structure. The absorption bands observed around 3300–3500 cm^− 1^ are associated with the stretching vibrations of N–H and O–H groups. PAMAM and PAMAM-ZnO show broad peaks in this region, attributed to the stretching vibrations of hydroxyl (O-H) and amine (N-H) groups. However, the intensity of the O-H/N-H peak in PAMAM-ZnO is reduced compared to PAMAM. This indicates that some hydroxyl or amine groups in PAMAM are involved in interactions with ZnO, likely through coordination or hydrogen bonding. The broadening of the spectrum in this region is attributed to the presence of O–H groups and the N–H stretching within the PAMAM structure. The N–H groups came from absorption of amide and amine of terminated groups (H_2_N-(CH_2_)_2_-NH-C = O) are particularly significant as they play a crucial role in forming linkages with asphaltene particles, which contain a variety of functional groups, especially carboxylic groups. The N-H within PAMAM-ZnO can react with the carboxylic groups in asphaltene, forming an ether bond. This interaction is vital for enhancing the compatibility and bonding between the polymer matrix and the asphaltene particles, thereby improving the overall properties of the composite coating.

Further analysis revealed stretching bands around 2800–3000 cm^− 1^. Both spectra show peaks in this region corresponding to the C-H stretching vibrations of alkyl groups in PAMAM. The slight shift and reduction in intensity in PAMAM-ZnO could be due to the interaction between the polymer and ZnO nanoparticles. A strong peak is observed in both spectra near 1650 cm^− 1^, corresponding to the carbonyl (C = O) stretching vibrations from the amide bonds in the PAMAM structure. The intensity is slightly lower for PAMAM-ZnO, suggesting some degree of interaction between the carbonyl groups of PAMAM and the ZnO surface. A distinct feature of the PAMAM-ZnO spectrum is the appearance of new peaks in the lower wavenumber region around 500–600 cm^− 1^. These peaks are characteristic of Zn-O stretching vibrations, confirming the successful incorporation of ZnO nanoparticles into the PAMAM matrix.

**Fig. 2:**
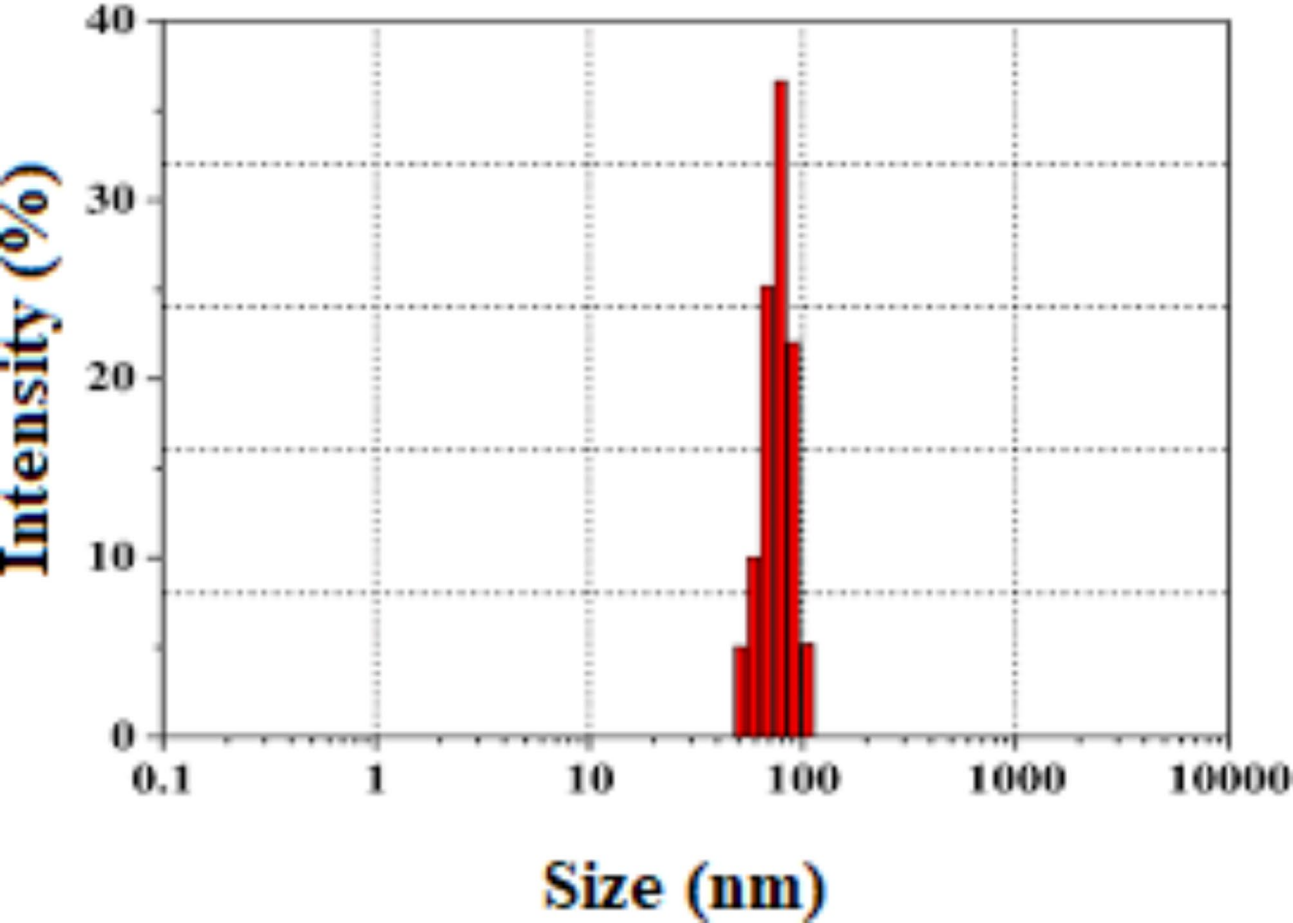
DLS image of PAMAM-ZnO.

The dynamic Light Scattering (DLS) analysis provides insights into the size and distribution of ZnO nanoparticles. Figure 2 represents the size distribution by number. The average hydrodynamic radius of the particles was determined to be 98 nm.

**Fig. 3:**
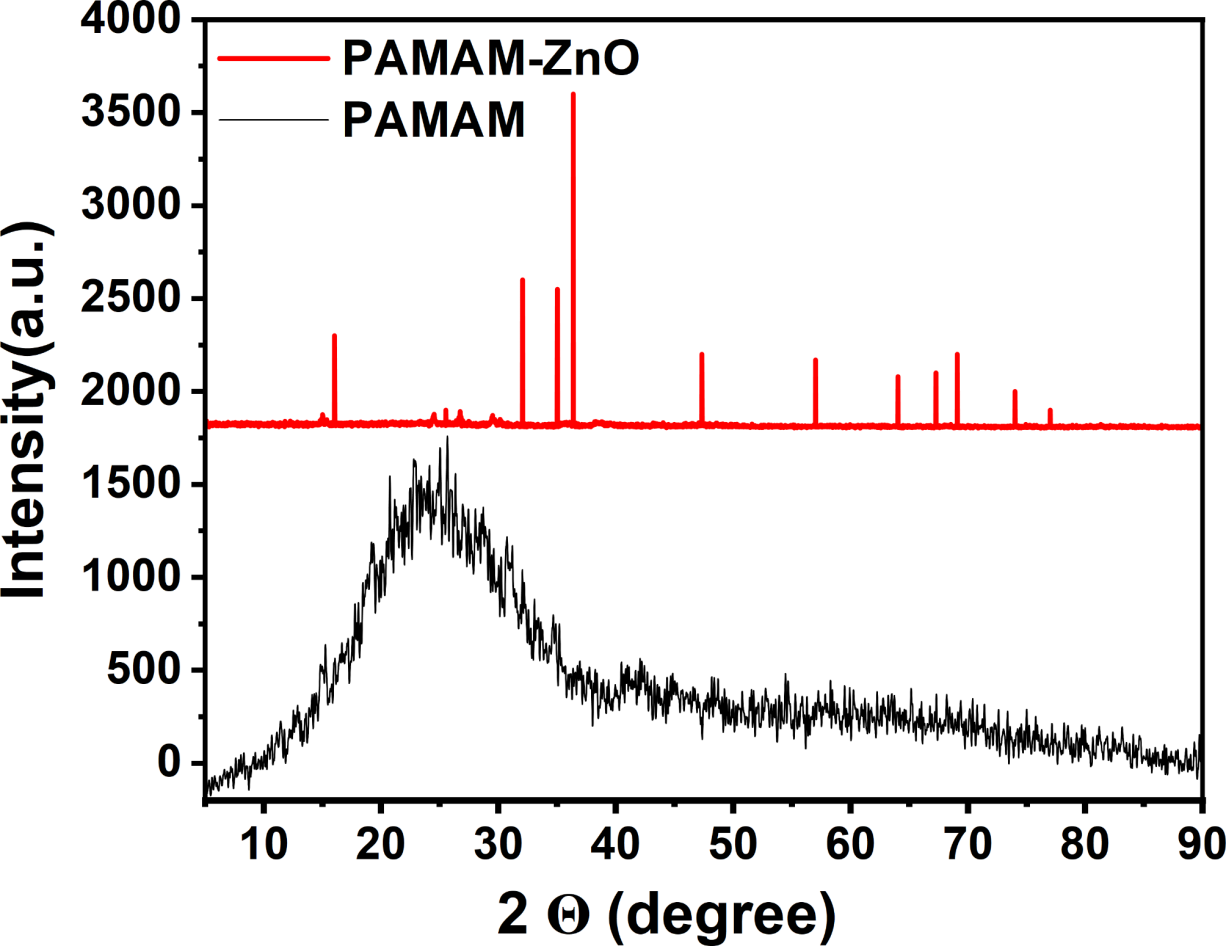
XRD patterns of PAMAM and PAMAM-ZnO nanocomposite.

The XRD analysis of PAMAM, ZnO, and PAMAM-ZnO nanocomposite materials was conducted, and the results are presented in Fig. 3. As indicated in Fig. 3, the XRD pattern of the PAMAM reveals the absence of peaks, except for a broad band occurring approximately at 2θ = 26°. This observation implies that the structure of the PAMAM is amorphous.

The X-ray diffraction pattern (2θ) of ZnO nanoparticles exhibits distinctive peaks at 31.66°, 34.58°, 36.12°, 47.76°, 56.5°, 62.7°, 66.32°, 67.76°, and 69.18° which are correlated of the (100), (002), (101), (102), (110), (103), (200), (112), and (201) crystal planes of hexagonal ZnO, respectively. as observed from Fig. 3. This may be perfectly indexed to the standard hexagonal wurtzite ZnO (ICDD No. 01-079-2205), which has lattice constants of a = 3.250 A˚, b = 5.207 A˚^36^. Furthermore, The diffraction pattern of the PAMAM-ZnO nanocomposite is identical to that of the ZnO nanoparticles, as shown in Fig. 3, providing conclusive evidence for the successful synthesis of PAMAM-ZnO nanocomposite. Notably, the sharp and well-defined peaks observed in Fig. 3 indicate the high crystallinity of the ZnO nanoparticles within the nanocomposite structure. This crystalline nature indicates the ordered arrangement of atoms within the ZnO crystal lattice, further affirming the quality and structural integrity of the synthesized ZnO nanoparticles in the PAMAM-ZnO nanocomposite. The determination of the average particle size (D) was carried out based on the Scherrer Eq. (1):

2 $${\text{D }} = {\text{ }}0.{\text{94}}\lambda /\beta {\text{Cos}}\theta$$

Here, λ signifies the wavelength ((1.5406 A˚), β represents the full width at half-maximum, and θ denotes the diffraction angle. The (101) plane is chosen to calculate the crystallite size. The particle size was determined using the Scherrer equation with the given parameters. The full width at half maximum of the ZnO (101) peak was 0.89 degrees, converted to 0.0155 radians. The Bragg angle for the (101) peak, corresponding to (2 theta = 31.66), was calculated as 15.83 degrees or 0.276 radians. Applying the Scherrer equation with the shape factor (k = 0.94) and the X-ray wavelength (λ = 1.51406 A˚), the particle size (D) was calculated to be approximately 95.7 nm. The discernible line broadening observed in the diffraction peaks implies that the synthesized materials fall within the nanometer range ≈ of 95.72 nm. Furthermore, the calculated lattice parameters align closely with the reported values. The reaction temperature notably influences the particle morphology of the ZnO powders prepared.

**Fig. 4:**
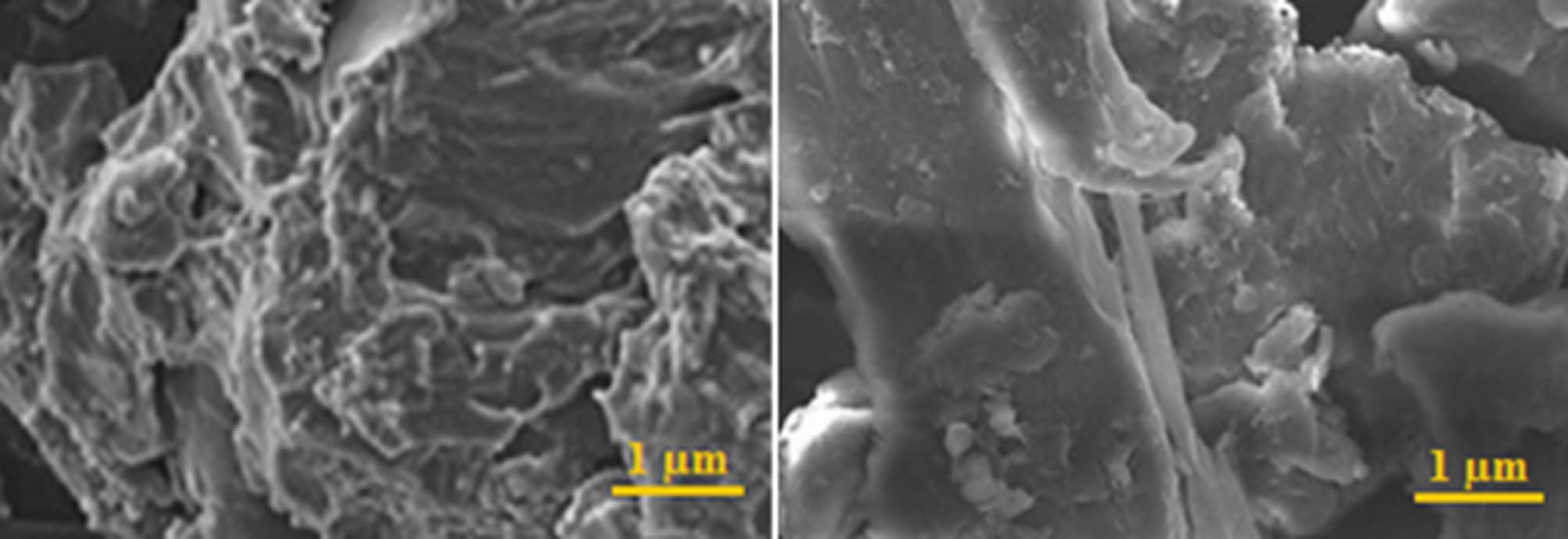
SEM images of the PAMAM-ZnO nanocomposite.

The SEM image of the PAMAM-ZnO nanocomposite reveals the complex structure of this nanohybrid material. Figure 4 shows the porous structure of PAMAM dendrimers and a combination of spherical dendritic structures and tiny ZnO particles woven together. These dendritic structures, representing the characteristic PAMAM dendrimer framework, show a clear and structured arrangement confirming the production process was carefully controlled. Figure 4 shows that the ZnO nanoparticles are evenly spread throughout the dendritic structure, indicating a uniform dispersion between these two materials. SEM image of PAMAM-ZnO reveals exciting shapes and sizes in the dendritic structures. ZnO varies from nano-scaled to micro-scaled, indicating different generations within the dendrimer structure. Additionally, it is worth noting that incorporating ZnO nanoparticles influences the surface roughness of the composite material. This microscopic depiction highlights the detailed structural complexities of the PAMAM-ZnO composite, emphasizing its potential for a wide range of uses.

**Fig. 5:**
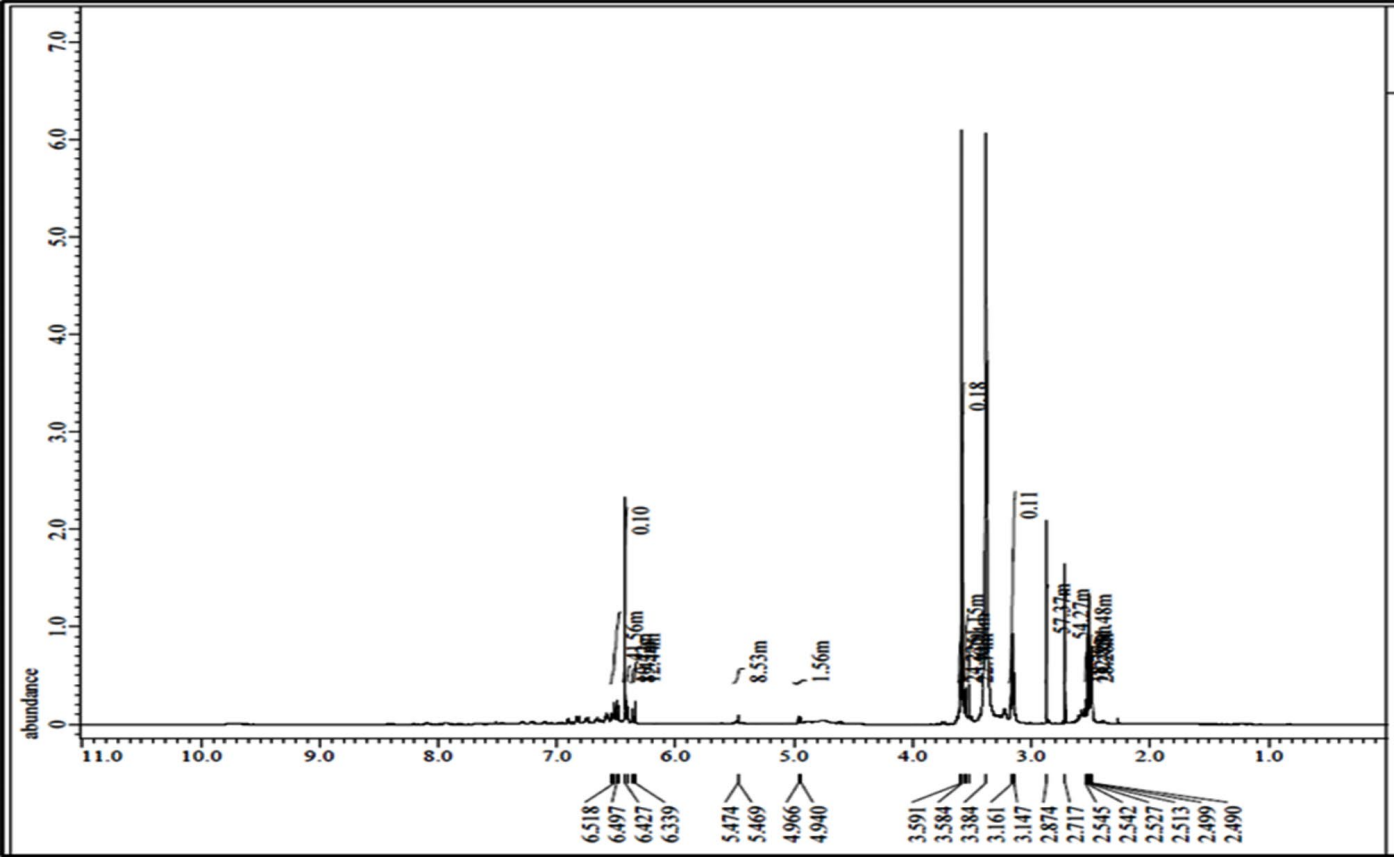
H-NMR spectrum of PAMAM-ZnO.

The structure of the prepared PAMAM was also confirmed by 1 H NMR spectroscopy, and the signal of each peak is represented in Fig. 5. Signal at δ about 8 ppm (NH–, 1), 2.2–3.6 attributable to the methylene protons of PAMAM [3.6 ppm (CH_2_–NH, 2), 3.4–3.2 ppm (CH_2_–NH, 3), 3 ppm (CH_2_–NH_2_, 4), 2.8 ppm (CH_2_–N, 5), 2.6 ppm (CH_2_–CH_2_–N–, 6), 2.5 ppm (CH_2_–C = O, 7), 2.2 ppm (NH_2_, 8).

### Mechanism of polymerization

The in-situ polymerization of PAMAM dendrimers on ZnO-APTES surfaces involves a controlled, stepwise process. First, ZnO nanoparticles are functionalized with APTES, introducing amine groups for further reactions. Methyl acrylate (MA) is then grafted onto the surface through Michael addition, modifying the ZnO to form ZnO-MA. Ethylenediamine (EDA) is slowly added at low temperature, initiating nucleophilic addition between EDA and the acrylate groups, leading to the growth of PAMAM dendrimers. The controlled temperature and prolonged reaction time allow for uniform dendrimer growth, forming a highly branched structure with terminal amine groups. The process concludes with centrifugation, washing, and drying, resulting in ZnO-PAMAM, which exhibits enhanced surface reactivity and is suitable for use in electrochemical anti-corrosive coatings due to the dendritic architecture.

The polymerization of PAMAM dendrimers is driven by nucleophilic addition, where ethylenediamine reacts with acrylate groups on the ZnO surface, forming amide bonds that serve as the structural backbone of the dendrimer. The dendritic structure results from the stepwise growth of multiple PAMAM layers, creating a highly branched, tree-like architecture with numerous terminal amine groups, which enhance the material’s reactivity and suitability for advanced coatings. Controlled temperature, prolonged reaction time, and a nitrogen atmosphere optimize the process, minimizing side reactions and ensuring uniform polymer growth on the ZnO surface without unwanted cross-linking.

### The prepared coating assessments

#### Physical-mechanical properties measurements of coating blends

The detailed physical and mechanical characteristics of various formulated coatings are presented in Table 3. The results indicate that the modified samples exhibit increased hardness compared to the pristine sample. There is a rise in softening point, specific gravity, and kinematic viscosities, accompanied by a decrease in penetration value. Additionally, the penetration index shows an increase with the addition and increment of PAMAM-ZnO from 2 wt% to 6 wt%. The modification of asphalt leads to improvements in both cohesion and elasticity. At elevated service temperatures (27 °C), the stiffness modulus of the polymer phase surpasses that of the matrix, contributing to increased viscosity. Conversely, at low temperatures, the stiffness modulus of the dispersed phase is lower than that of the matrix, mitigating brittleness. Consequently, the dispersed polymer phase enhances the engineering properties of asphalt, positively impacting viscosity, softening point, and toughness. The temperature sensitivity of the modified samples was checked by calculating the penetration index (PI) values. PI values increased from − 0.40 for unmodified asphalt to 0.999, 1.695, and 1.856 for 2, 4, and 6% of PAMAM-ZnO content, respectively. The changes in the hardness and stability of modified samples by PAMAM-ZnO were observed. These alternations are caused by PAMAM-ZnO forming a robust network with the asphalt, making it very viscous. It is now confirmed that PI is a direct indicator to classify the rheological behavior of asphalt as PI < 2 denotes asphalt gel while PI > 0 is typical in sol. It was observed that blown asphalt usually contained PI < 1 when straight asphalt had − 1 > PI > + 1. Only sensitive materials, such as coal tar, gave a PI > 1.

**Table 3:** Physical properties of pristine and 2, 4, and 6% PAMAM enriched ZnO modified asphalt.

Characteristics	Pristine asphalt (AC)	Modified asphalt binder			
		Asphalt + PAMAM-ZnO			
		1%	2%	4%	6%
Penetration (at 25 ºC, 100 g, 5s) 0.1 mm	63	59	56	50	43
Softening point (ring and ball) ºC	49	54	59	65	67
Specific gravity (at 25 ºC)	1.03	1.04	1.08	1.14	1.18
Kinematic viscosity (at 135 ºC) c St	338	1200	1830	2050	2160
Penetration index (P.I)	− 0.40	0.255	0.989	1.685	1.945
Flash point	300	350	390	510	586
Heating point	445	500	546	610	680

#### Rheology measurements

**Fig. 6:**
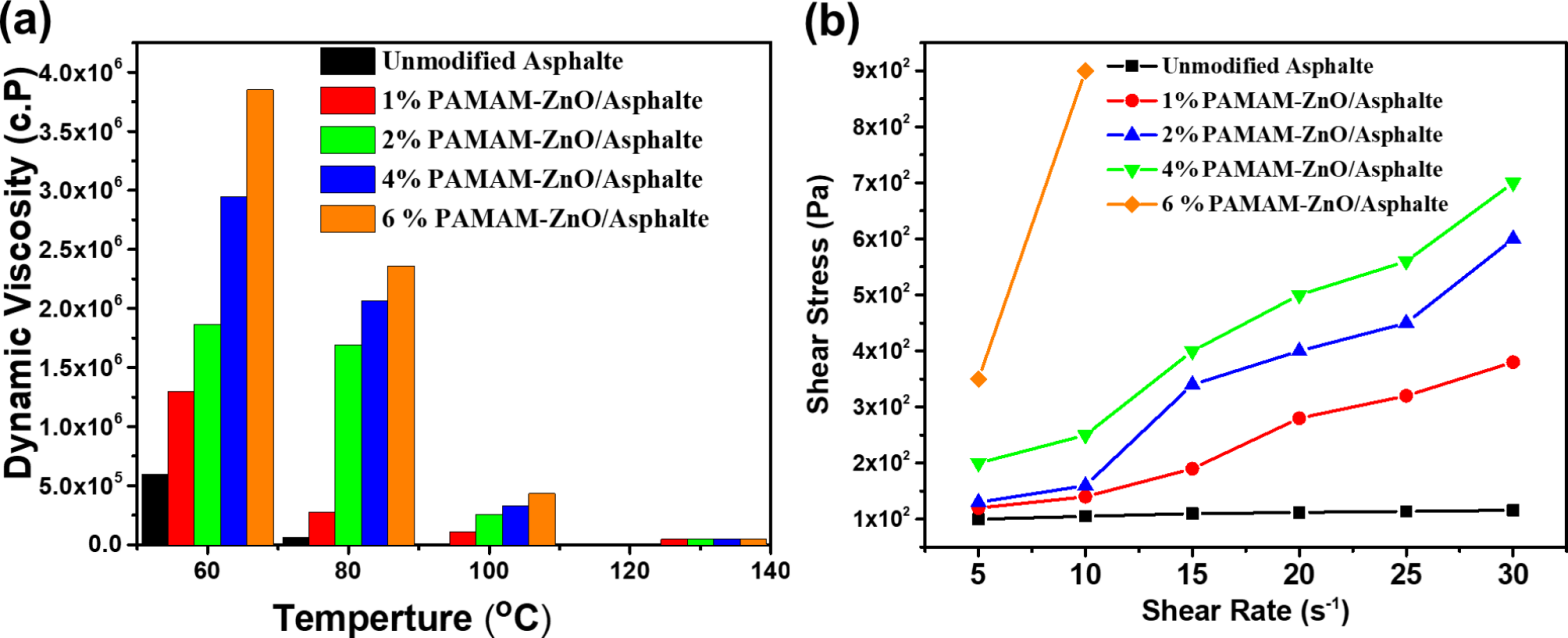
(**a**) The dynamic viscosity against temperature (**b**) shear stress and shear rate of unmodified and PAMAM-ZnO modified asphalt samples.

The viscosity of the unmodified sample and PAMAM-ZnO-modified asphalt samples against temperature is shown in Fig. 6a. As evident from the result, the polymer-modified asphalt (PMA) coatings showed a remarkable decrease compared to pristine asphalt. This decline is due to PAMAM particles, which improve the rheological properties of virgin asphalt. It is well known that dynamic viscosity decreases in the case of using RRNP& RNP at temperature 60 ^0^C in percentages of 1, 2, 4, and 6%w/w in percentages of 100, 200, 225, 250% & 300, 350, 360, and 600% respectively while at 120 ^0^C dynamic viscosity decreases in percentages 47, 50, 52& 53 and 20, 30, 43&45 respectively. The high differences between the two modifiers are due to amide groups in PAMAM, which interact with asphalt to form a very strong network and cause the asphalt to become highly viscous.

Another aspect of rheology that can be measured is shear stress (dyne/cm^2^) and shear rate (sec^− 1^). Measuring shear stress against shear rate reveals a fluid’s rheological properties, such as whether it is Newtonian or non-Newtonian. This information is crucial for predicting how the fluid will behave in various processes like mixing, pumping, and application, thus ensuring efficiency and product quality in pharmaceuticals and materials science^37^. As presented in Fig. 6b, the elasticity modulus of PMAs increases with increasing the polymer addition level in order of 1, 2, 4, and 6% w/w for PAMAM-ZnO. This phenomenon is attributed to PAMAM-ZnO functionality mainly reacting with carboxylic groups in asphaltenes, forming an ester link. This bond should prevent phase separation and improve storage stability.

Moreover, a chemical network could theoretically form if the asphaltene molecule, or micelle, contains more than one carboxylic group. Other reactions are possible between the PAMAM-ZnO terminated functional group and the functional groups present in asphalt; as previously mentioned, the amide group reacts with a carboxylic group to form an ether bond. Moreover, an intermolecular cross-linking reaction can occur once a reaction, water molecule, and hydroxyl group have been formed on the polymer main chain. That newly formed cross-bonding would lead to the forming of a polymer network that does not necessarily involve asphalt molecules.

#### Abrasion resistance

According to Table 4, PAMAM-ZnO samples have higher resistance than pristine samples due to strong reactions between carboxylic groups and asphaltene molecules, which form tougher samples. Also, increased additive content in the modified asphalt samples decreases weight loss. This result indicated that modifying asphalt using PAMAM-ZnO made it more abrasion-resistant than the commonly used 60/70.

**Table 4:** Abrasion resistance of PMAs with PAMAM-ZnO samples.

Sample type	wt. Before (gm)	wt. After (gm)	wt. Loss (gm)	wt. Loss % (gm)
Unmodified asphalt	79.3674	74.7674	4.6	5.7
1	79.0917	78.2305	0.8612	1.08
2	71.4425	70.6994	0.7431	1.04
4	70.916	70.2466	0.6694	0.94
6	73.5136	72.874	0.6396	0.87

### Evaluation of the prepared coating via electrochemical investigations

#### Open circuit potential

**Fig. 7:**
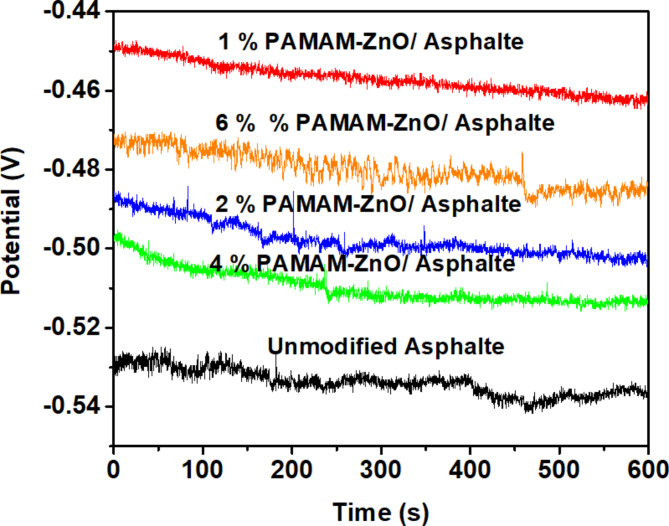
OCP of unmodified asphalt and formulated coatings with different compositions 1, 2, 4, and 6% PAMAM-ZnO/ asphalt in 1 M HCl.

The figure labeled as Fig. 7 illustrates the OCP values and their changes over time for both unmodified asphalt and asphalt doped with varying ratios of PAMAM-ZnO (1%, 2%, 4%, and 6%) in an acidic environment with a pH of 1.5 (1 M HCl). One noteworthy observation is that, within the first 15 min, the OCP values for the different PAMAM/ZnO-coated formulations shifted towards more positive potentials compared to pure asphalt. The OCP values for various coated carbon steel samples can be shown in Table 5. This indicates improved carbon steel surface protection against the corrosive ions, OH^−^, Cl^−,^ and O_2_ penetration. However, it is essential to note that the extent of this positive shift in the corrosion potential varies among the different formulated asphalts’ coatings. This variation in the OCP can be primarily attributed to the mitigation of the anodic reaction, which results from the improved protection of carbon steel provided by PAMAM-ZnO particles incorporated in these coatings^22^.

**Table 5:** OCP values for various carbon steel coated samples coated with unmodified asphalt and various formulated asphalt coatings in 1 M HCl.

Parameters	-E_corr_ (mV)
Unmodified asphalt	537.9
1%	462.7
2%	502.8
4%	508.1
6%	484.5

#### Tafel polarization

**Fig. 8:**
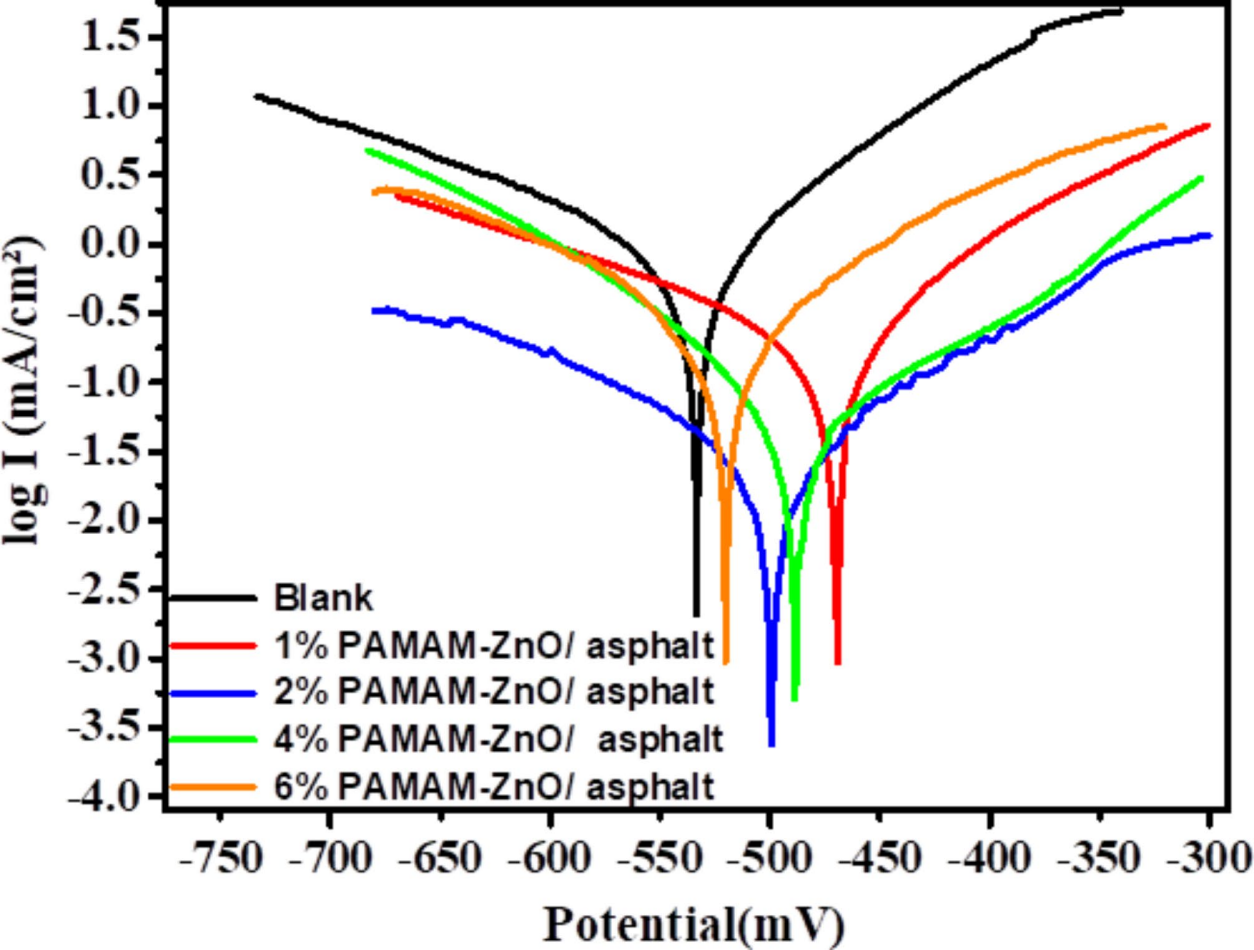
Tafel polarization of unmodified asphalt and formulated coatings with different compositions 1, 2, 4, and 6% PAMAM-ZnO/ asphalt in 1 M HCl.

Tafel measurements often initiate from a point distant from a stable state, which can introduce errors in the analysis. Potentiodynamic polarization records, for instance, commence at a potential approximately 200 mV below the OCP. Additionally, the determination of corrosion potential (E_corr_) from polarization curves is influenced by several variables, including the applied potential, current collection, and the duration of the polarization test^22^. This illustration indicates precisely why examining the system through EIS is a more reliable approach for anti-corrosive coatings. By inspection of potentiodynamic curves (Fig. 8), the semilogarithmic curves for both the anodic and cathodic arms can be observed to shift towards lower corrosion current densities (I_corr_). Also, the trend of E_corr_ shifted towards more positive values compared to the blank asphalt sample. So, these behaviors of different formulated coatings affirmed that adding the synthesized composite influenced the corrosion protection process via a mixed-type mechanism^38^. The polarization measurements were used to calculate kinetic vital parameters such as E_corr_, I_corr,_ cathodic and anodic Tafel slopes (βc and βa), and polarization resistance (R_p_). These calculated values are detailed and presented in Table 6. The formula provided below was utilized to compute the degree of coating protection efficiency (η%):

3 $$\:\varvec{\upeta\:}\mathbf{\%}\:=\frac{{CR}_{B}-{CR}_{S}}{{CR}_{B}}\:\times\:100$$

The CR_B_ and CR_S_ are the corrosion rates for the unmodified and the modified asphalt coatings under investigation conditions.

Furthermore, the Tafel plots revealed that the coatings synthesized using the optimal formulation have influenced the metal dissolution mechanism. The diminished metal erosion by the inserted medium is evident from the notable shift in the slopes of the cathodic and anodic Tafel lines towards more positive values. This slight swing of the anodic branch confirms the effective dispersion of PAMAM-ZnO within the asphalt matrix, which acts to passivate metal dissolution through sacrificial protection provided by ZnO particles. Additionally, significant protection enhancement was achieved through the prevention of cathodic reactions. The shift of βc from 196.7 mV to 87.9 confirmed the formation of a robust barrier layer on the metal surface, effectively suppressing hydrogen evolution at the WE due to secondary amine groups in the PAMAM structure^39^. Based on these results, it can be inferred that a 2% ratio of PAMAM-ZnO/asphalt exhibited the most substantial corrosion protection efficiency, equaling 97.93% compared with other formulations under investigation.

**Table 6:** The electrochemical key parameters of potentiodynamic polarization of unmodified asphalt and various formulated asphalt coatings in 1 M HCl.

Parameters	-E _corr_ (mV)	*R*_*p*_ (ohm)	I_corr_ (mA.cm^− 2^)	B_a_ (mV)	-Bc (mV)	C_*R*_ (mm.y^− 1^)	η (%)
Unmodified asphalt	533.5	28.55	0.8196	98.8	196.7	9.586	
1%	469.8	137.79	0.2150	96.1	164.2	2.514	73.77
2%	499.3	813.35	0.0171	92.3	87.9	0.198	97.93
4%	488.5	355.61	0.0919	116.7	109.0	1.108	88.44
6%	520.2	106.12	0.3610	137.5	171.3	4.222	55.96

#### Electrochemical Impedance Spectroscopy

**Fig. 9:**
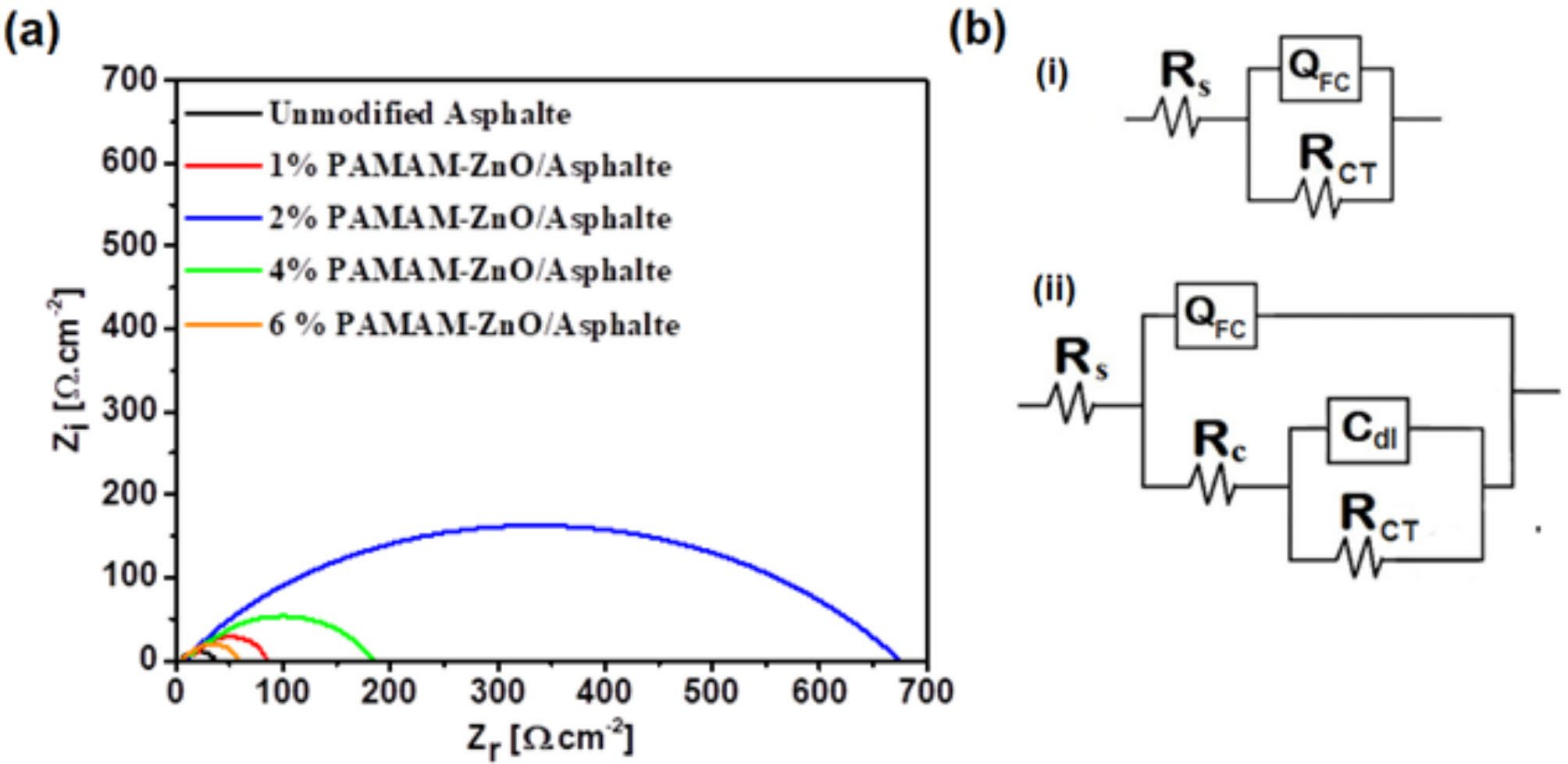
(**a**) Nyquist of EIS and (**b**) ECC of (i) unmodified asphalt and (ii) different formulated coatings with different compositions 1, 2, 4, and 6% PAMAM-ZnO/ asphalt in 1 M HCl.

Figure 9a displays the Nyquist plots obtained from the EIS experiments, revealing a substantial alteration in the carbon steel’s impedance response when exposed to the aggressive corrosive medium. The remarkable change revealed the impact of adding PAMAM/ZnO to the asphalt matrix. The EIS spectra of the different formulated coatings were fitted using EEC to obtain distinct evidence for how the fabricated coatings deal in the corrosive acidic medium. as depicted in Fig. 9b. The collected data were simulated using two appropriate EEC models: one consisted of Rs (Q_FC_ R_CT_), and the other involved Rs (Q_FC_ (R_FC_ (C_dl_ R_CT_)). The electrochemical parameters of the two different EEC are defined as Rs resistance hydrochloric solution, Q_FC_ refers to the formulated coating non-idea capacitance (constant phase elements), C_dl_ is a double-layer capacitance, R_FC_ signifies a formulated coatings resistance, and R_CT_ attributed to charge transfer resistance. The findings from these experiments, including the EIS parameters and the calculated corrosion protection efficiency (η%), have been summarized in Table 7. The polarization resistance (R_P_) was computed based on real impedance (Z_re_) data collected at both high and low frequencies, and the calculation method is detailed as follows^40^:

4 $$\:{R}_{P}={Z}_{re\:}\left(low\:ferquency\:\right)-\:{Z}_{re\:}\left(High\:ferquency\:\right)$$

Electrochemical C_dl_ was determined at the maximum frequency (*f*_max_), which corresponds to the point where the imaginary component of impedance reaches its maximum value, denoted as Z_max_. This calculation was performed utilizing the following equation^41^:

5 $$\:{C}_{dl}=\:\frac{1}{2\:{}_{max}}\:-\:\frac{1}{{R}_{CT}}$$

Ultimately, the η% was determined based on the values of R_P_ using the subsequent equation:

6 $$\:\:\%=\frac{{R}_{p\:}\left(\:modified\:asphalt\right)-\:{R}_{P\:}\left(\:unmodified\:asphalt\right)}{{R}_{P\:}\left(\:modified\:asphalt\right)}\times\:100$$

Where R_p_ is polarization resistance, and it was equal (R_P_ = R_FC_+R_CT_).

Upon observing the Nyquist plots for different samples displayed in Fig. 9a, it is noteworthy that the simulated circuit for unmodified asphalt corresponds to a single loop. In contrast, when the PAMAM-ZnO composite was introduced into the asphalt matrix with various ratios, the resulting fitted circuit exhibited a distinctive feature with two loops. As is known, the protective coating hindered the corrosive molecules through the barrier effect, and rheology assisted in the confirmation of this phenomenon, which means the coating film lengthened the path of the aggressive ions to the metal surface^16,22^. In the case of unmodified asphalt, the barrier effect is significant in the role of carbon steel protection, which is evident in the R_FC_ term. So, the one EEC component of the impedance response expressed the resistance ability of pristine asphalt to protect the metal surface.

**Scheme 3:**
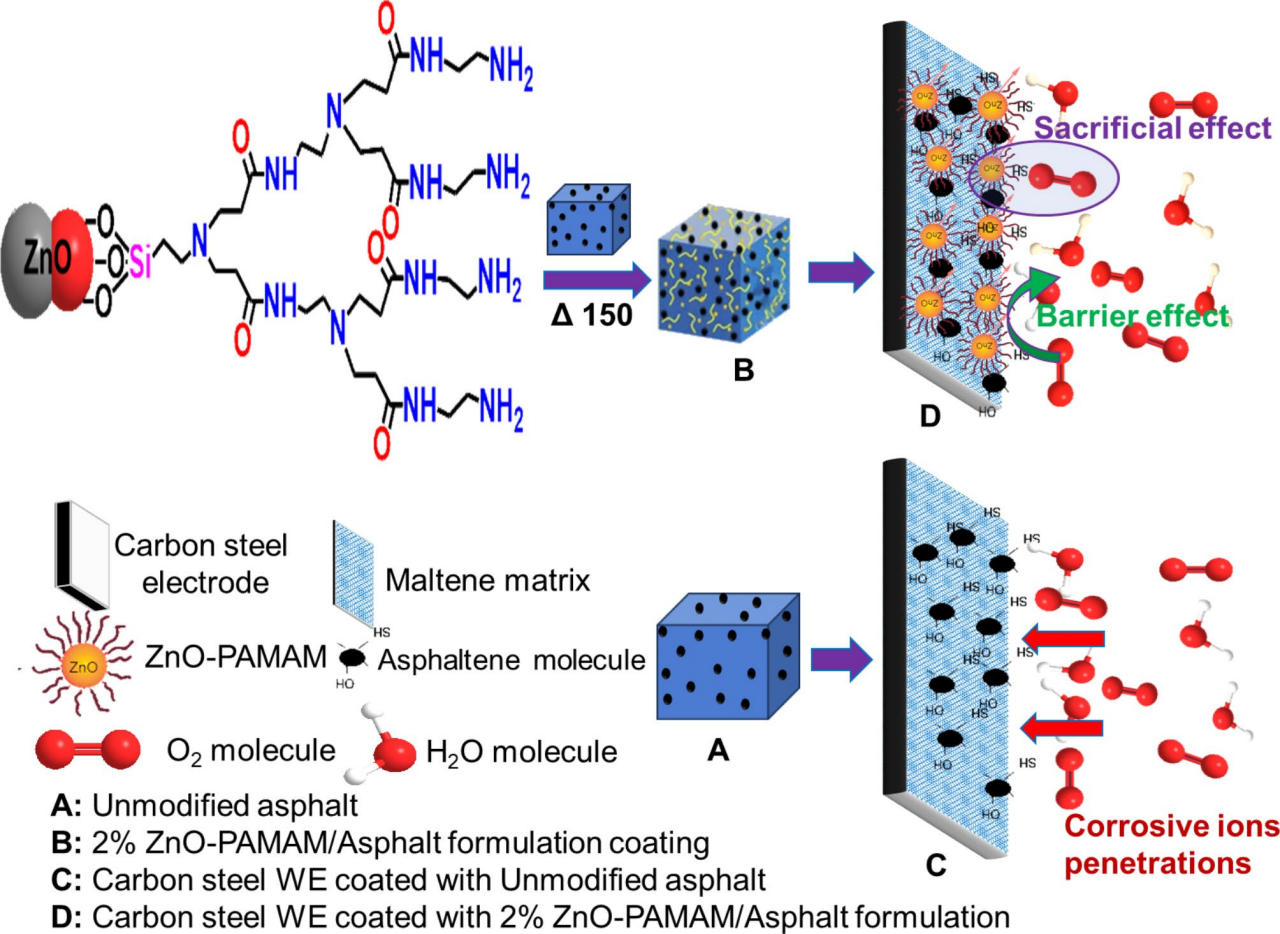
Both barrier effect enhancement and passivation influence for carbon steel corrosion hindering through the 2% PAMAM-ZnO/asphalt formulation coating.

Regarding the modified asphalt with varying ratios of PAMAM-ZnO, the presence of an additional EEC component observed can be attributed to the passivation effect, which is another vital factor that significantly influences the effectiveness of protective coatings, and it can be expressed in R_CT_ values. This effect involves inhibiting metal dissolution through coating additives, ZnO particles, that dissolve in the corrosive medium instead of the metal atoms. This mechanism underlies how passivation can impact the metal surface^40^. In Scheme 3, the efficacy of enhancing the barrier effect and the impact of passivation on carbon steel corrosion protection have been demonstrated. The particular EIS module presented by modified asphalt samples results from the passivation capability of ZnO molecules. The introduction of ZnO decreased the corrosion rate (C_R_), as evident from the concurrent increase in the value of R_CT_. As clear from the results of EIS parameters, it is evident that inserting PAMAM into the asphalt matrix also significantly affects corrosion resistance through barrier defense. According to the physical-mechanical measurements and rheology assessment, the PAMAM-ZnO/asphalt with a 2% ratio enhanced the asphalt film quality, which means plugging the coated film pinholes, SEM-supported.

It also prevents the coating layer from cracking when applied to a metal surface, eventually promoting CS corrosion reduction^17,42^. Neither ratios lower nor higher than a 2% are insufficient to heal all the asphalt matrix defects. Ethier higher or lower ratios of PAMAM-ZnO led to nanoparticle agglomeration or produced coating film weak area, respectively. It is obvious that modifying the asphalt PAMAM-ZnO not only enhances the barrier but also the passivation performance. Comprehensively, the R_FC_ value increased from 31.47 Ω cm^2^ to 600.3 Ω cm^2^ for pristine and asphalt modified with 2% PAMAM-ZnO, respectively. This increase demonstrates the enhancement of the modified asphalt film’s barrier properties, resulting from reduced diffusion of corrosive ions at the metal/electrolyte interface^11^.

Furthermore, parallel to barrier effect improvement, the R_CT_ that contributes to assessing the corrosion resistance of the steel/electrolyte interface had also increased, reaching a maximum value of 75.91 Ω cm^2^ for the optimum ratio of PAMAM-ZnO/ asphalt. Both enhancements confirmed the effective roles played by PAMAM and ZnO, particularly when the adequate ratio of the composite is introduced into the asphalt matrix. Conversely, the findings also indicate that incorporating the PAMAM-ZnO composite into the asphalt matrix substantially decreased C_FC_ and C_dl_. This value drop signifies a reduction in the diffusion of electrolytes into the coating matrix and between the metal/asphalt interface, specifically through the areas with defects. In detail, the value of C_dl_ observed in the 2% PAMAM-ZnO/ loaded asphalt coating is notably lower compared to the other ratios. This finding suggests that the electric double-layer becomes thicker, reducing the current dielectric constant. Moreover, the substantial decrease in C_FC_ indicates a significant enhancement in the formulated coating’s resistance to water permeation^16^.

**Fig. 10:**
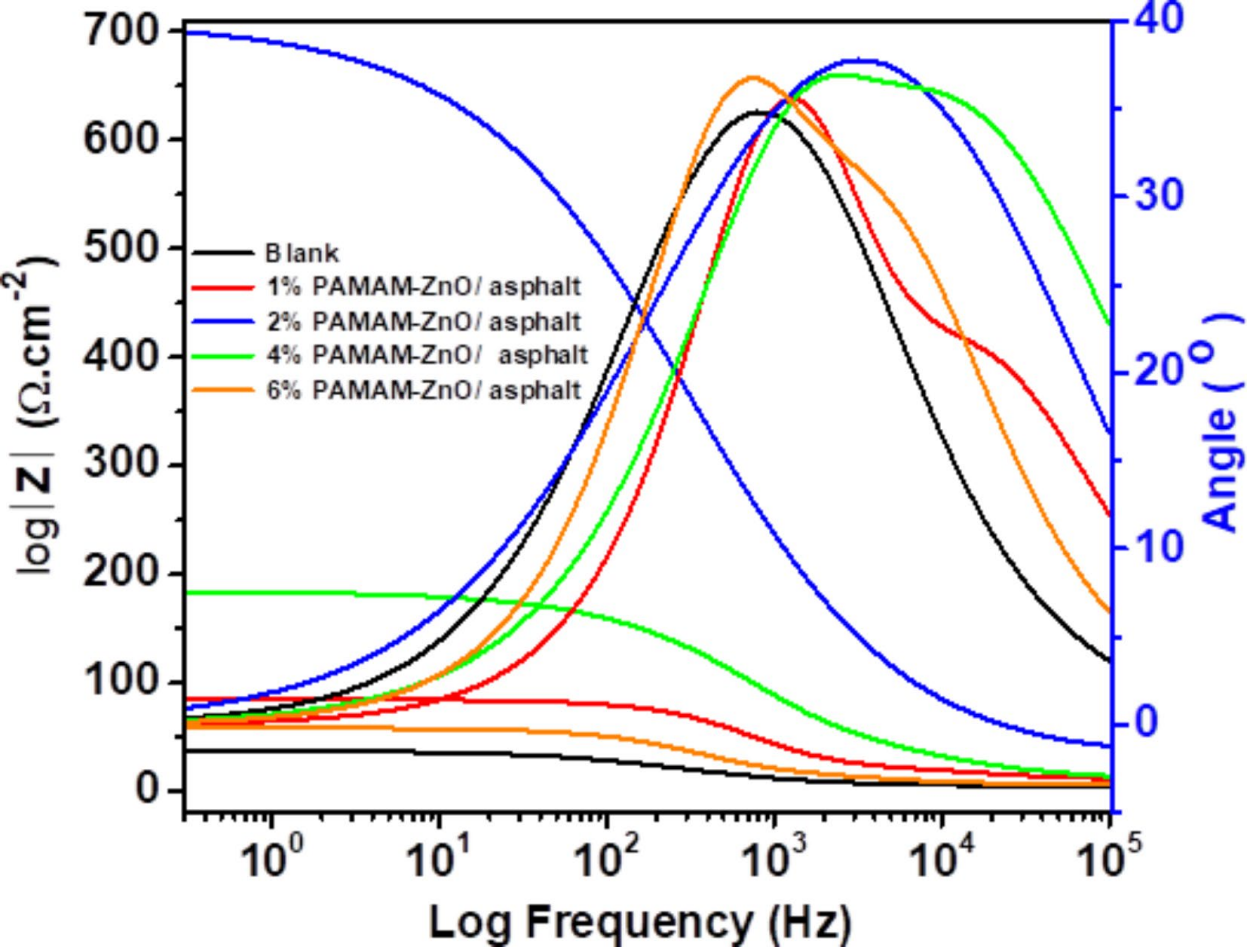
Bode plot of EIS for unmodified asphalt and formulated coatings with different compositions 1, 2, 4, and 6% PAMAM-ZnO/ asphalt in 1 M HCl.

While Nyquist plots provide valuable information regarding the investigation of coated metals’ resistance, as previously discussed, it is worth noting that Bode plot measurements offer greater reliability because the frequency dependence is discernible, ensuring that no information is lost^43^. The Bode phase curves corresponding to modified and unmodified asphalt are depicted in Fig. 10. The Bode phase plots exhibit two distinct time constants at high and low-frequency levels for all coated samples with the modified and pristine asphalt. These time constants are attributed to the R_FC_ and the C_dl_, respectively^11^. In cases where the PAMAM-ZnO/asphalt ratios exceeded 2%, there was a consistent decrease in the high-frequency (at 10 kHz) and R_P_. This decrease indicates a significant deterioration in the coating’s barrier properties, primarily due to the accumulation of composite particles within the asphalt matrix. This aggregation resulted in defects in the coating films, ultimately leading to the penetration of corrosive ions into the steel surface within these films. On the other hand, at low frequencies, the impedance value measured at 0.1 Hz was chosen as the parameter that represents the corrosion resistance of the coating. It can be observed that the |Z|_0.1 Hz_ expresses a dramatic change, increasing from 31.47 Ω cm^2^ to 680.54 Ω cm^2^ for both pristine asphalt and the optimum-formulated modified asphalt coatings. The phase angle of 40° of unmodified asphalt at high frequency, specifically |Z|_10_^3^_Hz_, indicates that the coatings exhibit a predominant non-ideal capacitive behavior, Q_FC_. In this context, it is essential to note that a phase angle of 0° signifies an ideal resistor, + 90° corresponds to a perfect inductor, and − 90° corresponds to an ideal capacitor. Values falling between these extremes may indicate non-ideal capacitance or mixed behavior, depending on the coating layer characteristics under investigation^44^. The shifts in the principal phase angle peak and the change in its intensity can be explained by modifications in the PAMAM-ZnO ratios present within the asphalt matrix. A minor distortion in the phase angle at a higher frequency, |Z|_10_^4^ Hz, attributed to C_dl_, has been observed for the modified asphalt when the PAMAM-ZnO content is above or below 2%. This deformation occurs due to the increase in the value of C_dl_, with the highest value obtained at 2.18E-04 F. cm^− 2^ for the 6% PAMAM-ZnO/asphalt formulation. Furthermore, a noteworthy lowest value of C_dl_ was obtained when the PAMAM-ZnO proportion was optimal, confirming that the electric double-layer became thicker while the local dielectric constant dropped.

According to EIS fitted data, the η% is maximized from 57.53 to 95.38% for 1 and 2% PAMAM-ZnO, respectively. This result proved the synergetic effect of both anti-corrosive effects, specifically the impact of the coating barrier, on the champion asphalt formulation to prevent corrosion. Moreover, it demonstrated the PAMAM’s ability to be an adequate linker into the asphalt matrix and boosted its capability to hinder the penetration of corrosive ions into the metals.

**Table 7:** The electrochemical key parameters of fitted EIS of unmodified asphalt and various formulated asphalt coatings in 1 M HCl.

Parameters	*R*_S_ (Ω.cm^− 2^)	Q_FC_ (F.cm^− 2^)	*n*	*R*_FC_ (Ω.cm^− 2^)	C_dl_ (F.cm^− 2^)	*R*_CT_ (Ω.cm^− 2^)	*R*_*P* (_Ω.cm^− 2^)	η %	RSE (%)
Unmodified asphalt	4.59	1.58E–04	0.74	31.47	–	–	–	–	7.314
PAMAM-ZnO/ asphalt ratio (%)									
1%	10.05	1.23E–03	0.76	35.62	3.21E–06	38.48	74.1	57.53	13.50
2%	9.21	1.29E–05	0.64	600.3	2.18E–04	80.24	680.54	95.38	8.55
4%	9.25	2.10E–05	0.65	98.9	1.38E–06	75.91	174.81	82.00	10.54
6%	7.68	3.59E–06	0.78	22.4	9.42E–06	29.9	52.3	39.83	8.29

## Conclusion

In conclusion, this study demonstrates the substantial enhancement of asphalt’s anti-corrosive properties through the incorporation of modified PAMAM with ZnO nanoparticles. The FTIR analysis reveals that the N–H groups in PAMAM are crucial for forming linkages with asphaltene particles, which are rich in carboxylic groups. This interaction significantly improves the performance of the asphalt coating. SEM imaging confirms the uniform dispersion of ZnO nanoparticles within the dendritic PAMAM structure, ensuring effective integration into the asphalt matrix. Physical and mechanical evaluations show that a 2% PAMAM-ZnO ratio enhances the cohesion and elasticity of the asphalt. EIS highlights the superior performance of the 2% PAMAM-ZnO formulation in mitigating asphalt matrix defects and providing exceptional corrosion resistance. This is supported by a corrosion protection efficiency of 97.93% from the Tafel test and a corresponding efficiency of 95.38% from EIS. The peak RCT of 75.91 Ω cm² underscores the formulation’s effectiveness in protecting carbon steel. Furthermore, the study underscores the dual corrosion inhibition mechanisms provided by the PAMAM-ZnO coatings. The barrier effect, combined with the passivation mechanism involving the dissolution of ZnO particles into the corrosive medium rather than the metal substrate, plays a crucial role in enhancing corrosion resistance. These results underscore the potential of PAMAM-ZnO-modified asphalt coatings in practical applications, offering substantial improvements in durability, corrosion resistance, and protection against environmental damage.

## Data Availability

The datasets used and/or analyzed during the current study are available from the corresponding author upon reasonable request.
